# New Point Mutations in Surface and Core Genes of Hepatitis B Virus Associated with Acute on Chronic Liver Failure Identified by Complete Genomic Sequencing

**DOI:** 10.1371/journal.pone.0123139

**Published:** 2015-04-07

**Authors:** Hangdi Xu, Mingfei Zhao, Guohua Lou, Min Zheng, Qingyi Cao, Zhi Chen

**Affiliations:** 1 Sir Run Run Shaw Hospital, Zhejiang University, Hangzhou, Zhejiang, China; 2 Department of Neurosurgery, Second Affiliated Hospital, School of Medicine, Zhejiang University, Hangzhou, Zhejiang, China; 3 State Key Laboratory for Diagnosis and Treatment of Infectious Diseases, Institute of Infectious Diseases, First Affiliated Hospital, School of Medicine, Zhejiang University, Collaborative Innovation Center for Diagnosis and Treatment of Infectious Diseases, Hangzhou, Zhejiang, China; Centro de Investigación en Medicina Aplicada (CIMA), SPAIN

## Abstract

The objective of this study was to identify new viral biomarkers associated with acute on chronic liver failure (ACLF) by complete genomic sequencing of HBV. Hepatitis B virus mutations associated with ACLF were screened by Illumina high-throughput sequencing in twelve ACLF cases and twelve age-matched mild chronic hepatitis B patients, which were validated in 438 chronic hepatitis B patients (80 asymptomatic carriers, 152 mild chronic hepatitis B patients, 102 severe chronic hepatitis B patients and 104 ACLF patients) by direct sequencing. The results of Illumina sequencing showed that the mutations at 7 sites (T216C, G285A, A1846T, G1896A, C1913A/G, A2159G, and A2189C) of 12 ACLF patients were significantly higher than those of 12 controls. In the validation cohorts, a significantly higher ratio of genotype B to C was found in patients with ACLF than in patients with non-ACLF. Multivariate analysis showed that T216C, G1896A, C1913A/G and A2159G/C were independent risk factors for ACLF. C216 in any combination, A/G1913 in any combination, and G/C2159 in any combination had high specificity for ACLF. In summary, T216C and A2159G/C mutations were novel factors independently associated with ACLF. Combined mutations in hepatitis B cases could play important roles in ACLF development.

## Introduction

Infection of hepatitis B virus (HBV) is one of the most serious and prevalent global health problems. More than 350 million people worldwide are chronically infected with HBV [1]. HBV related acute on chronic liver failure (ACLF) is a main cause of death for patients with chronic hepatitis. The mortality of ACLF is 60–80%, resulting in 22,600 deaths a year [2]. In addition to host factors, viral factors per se could also play an important role in determining clinical outcomes of chronic hepatitis B [3].

HBV is a small enveloped DNA virus of the Hepadnaviridae family with approximately 3200 nucleotides. Since HBV replicates through the reverse transcription of pregenome RNA, mutations occur more frequently in HBV than in other DNA viruses [4]. Therefore, mutations in the HBV genome may accumulate with time, and some of them might serve as viral markers for predicting the development of HBV-associated liver failure. Recently, several studies have suggested that patients with basal core promoter (BCP) mutations (T1762/A1764) had higher risks of ACLF [5, 6]. However, BCP mutations are also frequently found among patients with mild chronic hepatitis B (CHB-M) and asymptomatic carriers (ASC). In addition to these two mutations, mutations at nt.1846, nt.1896, and nt.1913 have also been found to be associated with the development of ACLF [7]. All of these studies focused mainly on the particular portions of the HBV genome (e.g. BCP and precore (PC) regions), and rarely analyzed the other locus that might play important roles. Hence, it remains unclear whether other predictive markers might be found by comparative analysis of the complete HBV genomes between different stages of liver disease. The aims of present investigation were to screen new variants of HBV related to the risk of ACLF development which had been ignored before and to find some specific biomarkers in the HBV genome for chronic hepatitis B progression. In present study, the whole-genome sequencing of HBV isolated from twelve CHB-M patients and twelve age-matched ACLF patients was conducted, and the mutations of HBV related to ACLF were screened by Illumina high-throughput sequencing. In addition, an independent case-control study with 438 cases was carried out to verify the association between the newly identified mutations in HBV genome and serious hepatitis B by direct sequencing.

## Materials and Methods

### Subjects

All of the patients in present study were from the southeast of China. The diagnosis criteria were based on the Management Scheme of Diagnostic and Therapy Criteria of Viral Hepatitis issued by Chinese Society of Infectious Diseases and Parasitology and the Chinese Society of Hepatology [8]. All participants were positive for HBsAg for at least 6 months, while subjects with hepatitis C/D, hepatocellular carcinoma (HCC), human immunodeficiency virus and other metastatic liver diseases were excluded in present study. The included ASC patients met the following criteria: (1) normal serum alanine aminotransferase (ALT) (no more than 45 U / l); (2) no evidence of liver cirrhosis based on the pathological or ultrasound examination. The CHB-M group consisted of CHB patients with mild liver disease symptoms or without liver disease symptoms. The ALT in CHB-M group was ≤120U/L, and plasma prothrombin activity (PTA) values were normal. The diagnosis of severe chronic hepatitis B (CHB-S) had to meet at least one of the following criteria: (1) serum albumin level ≤32 g/L; (2) serum total bilirubin (TBIL) values exceeding five-times the upper limit of normal (17.1 umol/L).; (3) PTA between 40% and 60%; (4) serum cholinesterase≤4,500 IU/L. The diagnosis of ACLF was according to the criteria defined by the Asian Pacific Association for the Study of the Liver [9]. Briefly, acute hepatic injury manifestated as increasing jaundice (serum TBIL>85 umol/L) and coagulopathy (PTA <40%) in patients with previously diagnosed or undiagnosed chronic liver diseases, complicated by ascites and/or encephalopathy within 4 weeks. Twelve ACLF patients and twelve CHB-M patients were recruited for full-length genome analysis with Illumina high-throughput sequencing. There were no significant differences of age, sex and genotype distribution between two groups. The plasma HBV DNA levels of these patients were >100,000 copies/mL. There was not any patient with pre-existing liver cirrhosis or a history of receiving antiviral treatment. The mutations screened by full-length genome analysis were validated in 438 patients with chronic HBV infection (80 ASC, 152 CHB-M patients, 102 CHB-S patients and 104 ACLF patients). There were the intact clinical and serological data for each subject. The present study was approved by the Ethics Committee of Zhejiang University. All subjects were informed of the objective of this study and signed the informed consent.

### PCR amplification and Illumina high-throughput sequencing

HBV DNA was extracted from 200 uL serum using a QIAamp DNA blood mini kit (Qiagen, Germany), according to the manufacturer's instructions. Primers pairs F1-R1, F2-R2, and F3-R3 were used to amplify 3 overlapping HBV full-length fragments (Table 1). PCR reactions were performed in a 50 μL reaction mixture that contained 2 uL extracted DNA, 1×Prime Star buffer (Mg2+Plus), 0.2 mM dNTPs, 0.5 μM forward primer, 0.5 μM reverse primer, and 2.5 U Prime Star (TaKaRa Bio, Shiga, Japan). The conditions used for PCR were 1 cycle of 95°C for 3 min followed by 45 cycles of denaturation for 30 s at 95°C, annealing of primers for 15 s at 57°C, and extension for 2 min with a final 10 min extension at 72°C. PCR products were utilized for the high-throughput sequencing, which was carried out on a Genome Analyzer II platform (Illumina, San Diego, CA, USA). Briefly, DNA was fragmented to smaller than 500bp using a sonicator device. A DNA library with a median size of 350bp±50 bp was prepared by ligating adapters to the fragment ends to generate flow-cell-suitable templates. The adapter-modified DNA fragments were pooled and attached to the flow cells by complementary surface-bound primers, and then isothermal bridging amplification formed the multiple DNA clusters for reversible terminator sequencing [10–12].

**Table 1:** Primers used for Illumina sequencing and direct PCR sequencing of HBV.

Primer	Sequence (5’—>3’)	Position
F1	AGTGGGCCTCAGTCCGTTT	645–663
R1	AAAAAGTTGCATGGTGCTGGTG	1804–1825
F2	AACGACCGACCTTGAGGCATACTTC	1685–1709
R2	GTTCCCAAGAATATGGTGACCC	2814–2835
F3	AATCTCGGGAATCTCAATGTTAG	2430–2452
R3	AGGGACTCAAGATGTTGTACAGACT	764–788
F4	ATGCAACTTTTTCACCTCTG	1814–1833
R4	GTAAAGTTTCCCACCTTATG	2466–2485
F5	GGAGCGGGAGCATTCGG	3022–3038
R5	ATAAAACGCCGCAGASACA	378–396
F5.1	TCCTAGGACCCCTGCTCGTGTTAC	177–200

The error of Illumina high-throughput sequencing is higher than that of the Sanger method, therefore, a plasmid DNA containing full complete HBV served as an error rate control for measuring the accuracy of the Illumina high-throughput sequencing. The control plasmid and 24 samples were prepared simultaneously for Illumina high-throughput sequencing.

Illumina reads from the control plasmid were mapped using the Bowtie software, with the default parameters. An initial analysis revealed that the error rate varied as a function of position within the read (S1 Fig), so only the first 20 nucleotides of each short reads were aligned at last, allowing mismatch with 2 nucleotides. Illumina reads from twelve ACLF and twelve CHB-M patients were also aligned using BOWTIE software, referencing to sequences of Chinese HBV genotype B/ genotype C [13].

The overall error rate of plasmid control sequence was 0.49%, and the mean number of nucleotides with ≥4% mismatch error rate per one hundred nucleotides was 2.4. According to this result, 4% was empirically served as the limit to distinguish the authentic variants from technological artifacts [14].

### Analysis of HBV mutations by direct sequencing

Semi-nest PCR was used to amplify the two target regions of HBV genome. Primer pairs F2-R4 were used as the first-round primers, and primer pairs F4-R4 served as the second-round primers for regions A. Primer pairs F5-R5 were utilized as the first-round primers, and primers-pairs F5-R5.1 served as the second-round primers for regions B (Table 1). PCR was performed under the conditions described above except that the elongation time was changed to 1 min and the cycles were reduced to 25.

### Examination of serological HBV markers, HBV DNA concentration and HBV genotyping

HBV serum markers were tested by commercial enzyme immunoassay kits (Abbott AxSYM System, Germany). HBV DNA was quantified by a PCR diagnostic kit (PG Biotech, Shenzhen, China) with a detection limit of 500 copies /mL. HBV genotypes were detected by a multiplex PCR assay [15].

### Statistical analyses

Chi-square test or Fisher’s exact test was used to determine the differences in categorical variables. Continuous variables with normal distribution were tested by one-way ANOVA among multiple samples and by Student’s t-test between two samples. Continuous variables with skewed distribution were compared using Kruskal-Wallis test among multiple samples and with the Mann-Whitney U-test between two samples. Serum viral loads with skewed distribution were adjusted to normal distribution by transformation into logarithmic function, and then tested by Student’s t-test or one-way ANOVA. Forward stepwise multivariate regression analysis was performed to determine the risk factors associated with ACLF development. All statistical tests were two sided, and performed using the Statistical Program for Social Sciences 13.0 (SPSS13.0). *P* value of < 0.05 was considered as statistically significant. The combined mutation frequencies of HBV were estimated by SHEsis software platform (a platform for analyses of haplotype construction) [16].

## Results

### Results of Illumina high-throughput sequencing

Table 2 lists the clinical data and mutations characteristics of twelve serum samples from ACLF cases and twelve serum samples from CHB-M cases. There were no differences of age, sex, HBV DNA level, and genotype distribution of HBV between ACLF and CHB-M groups. In ACLF group, the mutations at seven sites (T216C, G285A, A1846T, G1896A, C1913A/G, A2159G and A2189C) were frequently found, and there were significant differences in the mutation frequencies at these sites between the ACLF and CHB-M groups (*P*<0.05 or *P*<0.01).

**Table 2:** Clinical characteristics and HBV mutation features of the patients involved in Illumina sequencing.

Characteristics	ACLF(n = 12)	CHB-M (n = 12)	*P*-value
Age (mean±SD)	35±6.12	35.7±7.58	NS
Sex (male/female)	11/1	10/2	NS
HBV genotype (B/C)	10/2	8/4	NS
HBV DNA (log10 copies/ml)	6.71±1.54	6.74±1.19	NS
HBeAg positive rate	4/8	10/2	<0.05
ALT (U/liter)	648.7±728.1	77.5±38.3	<0.01
AST (U/liter)	376.2±453.9	65.0±32.8	<0.01
Total bilirubin (mg/dl)	324.9±129.3	15.6±12.7	<0.01
Mutated /unmutated samples			
T216C	10/2	1/11	—
G285A	9/3	1/11	—
A1846T	10/2	7/5	—
G1896A	10/2	7/5	—
C1913A/G	8/4	4/8	—
A2159G	10/2	5/7	—
A2189C	11/1	6/6	—
Virus mutations frequencies (%, Mean±SD)			
T216C	58.9±44.5	2.58±4.76	<0.01
G285A	44.8±44.9	1.33±2.06	<0.01
A1846T	71.9±37.5	22.67±33.1	<0.05
G1896A	63.4±42.1	29.6±39.3	<0.05
C1913A/G	47.6±42.8	15.2±24.7	<0.05
A2159G	71.7±41.7	13.4±29.0	<0.05
A2189C	78.6±36.7	18.3±30.0	<0.05

ALT, alanine aminotransferase; AST, aspartate aminotransferase; —,not calculated; ACLF, acute on chronic liver failure; CHB-M, mild chronic hepatitis B; NS, not significant.

### Results of HBV gene mutations detected by direct sequencing at different stages of liver disease

The clinical and virological characteristics of 438 cases are showed in Table 3. The frequency of HBeAg expression in ACLF group was significant lower and the ratio of genotype B to C in ACLF group was significantly higher than those in other three groups (*P*<0.01). The nucleotide sequences have been submitted to the GenBank databases under the accession numbers JX681227-JX681664 and JX847884-JX848321. The mutation frequencies at seven sites in ACLF group enhanced significantly, as compared with ASC, CHB-M, and CHB-S groups (*P*<0.05). The mean values of substitutions at seven sites in ASC, CHB-M, CHB-S and ACLF groups were 0.88, 1.38, 2.34 and 3.74, which increased in a stepwise manner.

**Table 3:** The clinical data and HBV mutation profiles in 438 subjects.

Clinical data and mutation rates	ASC (n = 80)	CHB-M (n = 152)	CHB-S (n = 102)	ACLF (n = 104)
Age (mean ± SD)	38.5±11.4	37.6±10.9	40.7±13.2^e^	40.9±11.5^e^
Sex (male/female)	58/22	112/40	74/28	91/13^d^ ^,^ ^b^ ^,^ ^c^
HBV genotype(B/C/D)	17/61/2	22/130	29/73^b^	68/36^a^ ^,^ ^b^ ^,^ ^c^
HBV DNA (log10 copies/ml, mean ± SD)	5.52±2.38	6.14±1.77^d^	5.77±2.05	6.09±2.05
HBeAg positive rate(+/-)	62/18	113/39	72/30	51/53^a^ ^,^ ^b^ ^,^ ^c^
ALT (U/liter, mean ± SD)	31.8±12.6	79.5±78.7^a^	270.6±414.3^a^ ^,^ ^b^	310±432.8^a^ ^,^ ^b^ ^,^ ^f^
AST (U/liter, mean ± SD)	28.1±9.9	49.8±38.2^a^	175.9±251.3^a^ ^,^ ^b^	213.0±231.8^a^ ^,^ ^b^ ^,^ ^c^
Total bilirubin (mg/dl, mean ± SD)	13.4±5.0	14.9±7.6	98.3±114.4^a^ ^,^ ^b^	317.5±160.3^a^ ^,^ ^b^ ^,^ ^c^
T216C (%)	7.5	9.2	22.5^a^ ^,^ ^b^	49.0^a^ ^,^ ^b^ ^,^ ^c^
G285A (%)	3.8	5.3	22.5 ^a^ ^,^ ^b^	44.2 ^a^ ^,^ ^b^ ^,^ ^c^
A1846T/G (%)	13.8	22.4	34.3^a^ ^,^ ^e^	58.7^a^ ^,^ ^b^ ^,^ ^c^
G1896A (%)	23.8	33.6	54.9^a^ ^,^ ^b^	72.1^a^ ^,^ ^b^ ^,^ ^f^
C1913A/G (%)	10	17.1	40.2 ^a^ ^,^ ^b^	55.8 ^a^ ^,^ ^b^ ^,^ ^f^
A2159G/C (%)	8.8	21.7^d^	24.5^a^	45.2^a^ ^,^ ^b^ ^,^ ^c^
A2189T/C (%)	21.3 ^a^	29.6	35.3^a^ ^,^ ^e^	49.0^a^ ^,^ ^b^ ^,^ ^f^
Substitutions (Mean ± SD)	0.88±1.32	1.38±1.68^d^	2.34±1.61^a^ ^,^ ^b^	3.74±1.52^a^ ^,^ ^b^ ^,^ ^c^

ALT, alanine aminotransferase; AST, aspartate aminotransferase; ASC, asymptomatic hepatitis B surface antigen carriers; CHB-M, mild chronic hepatitis B; CHB-S, severe chronic hepatitis B; ACLF, acute on chronic liver failure;

^a^ *P*<0.01, as compared with ASC.

^b^ *P*<0.01, as compared with CHB-M.

^c^ *P*<0.01, as compared with CHB-S.

^d^ *P*<0.05, as compared with ASC.

^e^ *P*<0.05, as compared with CHB-M.

^f^ *P*<0.05, as compared with CHB-S.

### Association between HBV mutations and the risk of advanced liver diseases in cases with genotype B virus and genotype C virus

In ACLF patients with genotype B virus, the substitution rates of T216C, G285A, A1846T/G, G1896A, C1913A/G, A2159G/C, and A2189T/C were 70.6%, 60.3%, 66.2%, 76.5%, 63.2%, 47.1%, and 50.0%, respectively; while in ACLF patients with genotype C virus, the substitution rates were 8.3%, 13.9%, 44.4%, 63.9%, 41.7%, 41.7%, 47.2%, respectively.

To avoid the potential influence on the results brought by age and sex differences among the 4 groups, we adjusted the age and sex in the flowing statistic analysis process. To clearly describe the associations of mutations in the S region and pre-core /core region of HBV genotypes B and C with ASC, CHB-M, CHB-S and ACLF, we firstly treated ASC as control (Table 4). Mutations at nt.216, nt.285, nt.1896, nt.1913 in genotype B were associated significantly with an increased risk of CHB-S and ACLF, after the adjustment for age and sex, respectively. In subjects with genotype C virus, mutation frequencies at nt.285, nt.1846, nt.1896, nt.1913, and nt.2159 were significantly higher in ACLF and mutation frequencies of nt.1846, nt.1896, nt.1913, nt.2159, and nt.2189 were significantly higher in CHB-S than those in ASC. As compared with patients with CHB-M, mutations at nt.216 and nt.285 were significantly associated with an increased risk of CHB-S in genotype B (Table 4), whereas mutations at nt.1896 and nt.1913 were significantly associated with an increased risk of CHB-S in genotype C. As compared with the patients with CHB-S, mutations at nt.2159 and nt.2189 in genotype B were significantly associated with an increased risk of ACLF, whereas there were no significantly differences at these 2 points at ACLF with genotype C (Table 4).

**Table 4:** The risks of CHB-M, CHB-S, and ACLF cases with 7 site mutations on the basis of genotype B and genotype C as compared with ASCs, CHB-M and CHB-S respectively.

	CHB-M*	CHB-S*	ACLF*	CHB-S**	ACLF**	ACLF***
Hotspot	AOR (95%CI)	AOR (95%CI)	AOR (95%CI)	AOR (95%CI)	AOR (95%CI)	AOR (95%CI)
Genotype B						
T216C	1.69 (0.24–11.94)	10.89 (2.03–58.45)^a^	21.06 (4.20–105.76) ^a^	5.34 (1.38–20.72)^b^	12.72 (3.21–50.46) ^a^	1.64 (0.65–4.12)
G285A	1.37 (0.18–10.54)	12.81 (2.29–71.61) ^a^	12.66 (2.54–63.14) ^a^	10.04 (2.07–48.75) ^a^	13.07 (2.66–64.15) ^a^	0.97 (0.39–2.40)
A1846T/G	3.75 (0.69–20.26)	3.42 (0.86–13.67)	6.84 (1.71–23.32) ^a^	1.35 (0.40–4.55)	2.37 (0.73–7.71)	2.09 (0.85–5.12)
G1896A	2.22 (0.51–9.67)	5.21 (1.39–19.56) ^b^	5.76 (1.79–18.49) ^a^	3.03 (0.89–10.30)	3.10 (1.04–9.20) ^b^	1.24 (0.45–3.39)
C1913A/G	1.79 (0.34–9.34)	5.45 (1.13–26.24) ^b^	8.12 (2.03–32.39) ^a^	2.64 (0.74–9.44)	5.94 (1.66–21.28) ^a^	2.21 (0.90–5.41)
A2159G/C	1.88 (0.09–38.71)	2.14 (0.19–23.53)	13.87 (1.72–112.15) ^a^	1.39 (0.19–10.47)	7.26 (1.48–35.65) ^a^	5.33 (1.66–17.08) ^a^
A2189T/C	3.64 (0.54–24.58)	1.71 (0.28–10.53)	7.19 (1.48–34.97) ^a^	0.52 (0.12–2.30)	1.63 (0.55–4.85)	3.67 (1.31–10.29) ^b^
Genotype C						
T216C	1.03 (0.30–3.52)	1.23 (0.32–4.57)	0.92 (0.17–4.86)	1.21 (0.41–3.61)	1.17 (0.28–4.84)	0.93 (0.21–4.13)
G285A	2.16 (0.23–20.11)	4.65 (0.56–42.57)	10.02 (1.076–93.39) ^b^	1.96 (0.49–7.79)	4.27 (1.01–18.13) ^b^	3.02 (0.75–12.25)
A1846T/G	2.88 (1.03–8.06) ^b^	4.61 (1.60–13.34) ^a^	7.52 (2.38–23.76) ^a^	1.63 (0.82–3.23)	2.73 (1.20–6.22) ^a^	1.74 (0.74–4.12)
G1896A	1.79 (0.86–3.74)	3.33 (1.54–7.22) ^a^	5.19 (1.95–13.80) ^a^	2.10 (1.15–3.84) ^b^	3.51 (1.57–7.86) ^a^	1.89 (0.81–4.43)
C1913A/G	1.97 (0.70–5.56)	7.01 (2.49–19.77) ^a^	7.53 (2.37–23.89) ^a^	3.72 (1.87–7.41) ^a^	4.95 (2.03–12.05) ^a^	1.15 (0.49–2.66)
A2159G/C	3.01 (1.17–7.75) ^b^	3.58 (1.32–9.66) ^a^	5.93 (1.98–17.74) ^a^	1.18 (0.61–2.28)	1.96 (0.87–4.38)	1.60 (0.68–3.77)
A2189T/C	1.56 (0.74–3.29)	2.49 (1.13–5.51) ^b^	2.41 (0.93–6.24)	1.61 (0.87–2.97)	1.86 (0.84–4.08)	1.15 (0.50–2.64)

ASC, asymptomatic hepatitis B surface antigen carriers; CHB-M, mild chronic hepatitis B; CHB-S, severe chronic hepatitis B; ACLF, acute on chronic liver failure; CI, confidence interval; AOR, adjusted odds ratio;

* compared with ASCs;

** compared with CHB-M;

*** compared with CHB-S.

^a^ *P* < 0.01, as compared with control.

^b^ *P* < 0.05, as compared with control.

As compared with non-ACLF cases, T216C, G285A, A1846T/G, G1896A, C1913A/G, A2159G/C, A2189T/C in genotype B and G285A, A1846T/G, G1896A, C1913A/G and A2159G/C in genotype C were significantly associated with an increased risk of ACLF(see Table 5).

**Table 5:** The risks of ACLF cases with 7 site mutations on the basis of genotype B and genotype C as compared with non-ACLF cases.

Hotspot	AOR (95%CI) in ACLF group	
	Genotype B	Genotype C
T216C	4.35 (2.08–9.11)^a^	0.95 (0.25–3.57)
G285A	2.88 (1.39–5.95) ^a^	4.28 (1.27–14.38)^b^
A1846T/G	2.76 (1.34–5.72) ^a^	2.75 (1.30–5.80) ^a^
G1896A	2.49 (1.17–5.31) ^b^	3.00 (1.43–6.31) ^a^
C1913A/G	3.69 (1.78–7.65) ^a^	2.87 (1.36–6.06) ^a^
A2159G/C	7.04 (2.78–17.87) ^a^	2.21 (1.05–4.62) ^b^
A2189T/C	3.35 (1.55–7.21) ^a^	1.63 (0.79–3.37)

ACLF, acute on chronic liver failure; CI, confidence interval; AOR, adjusted odds ratio.

^a^ *P* < 0.01, as compared with non-ACLF.

^b^ *P* < 0.05, as compared with non-ACLF.

### Risk factors independently associated with ACLF as compared with non-ACLF

Patients’ age, sex, HBV genotypes, HBV DNA level (log10 copies/ml), HBeAg status, and the presence of mutations at seven sites were included in the multivariate regression model (Table 6). It was found that the old age, genotype B, T216C, G1896A, C1913A/G and A2159G/C mutations were risk factors independently associated with ACLF, as compared with non-ACLF (*P* < 0.05 or *P* < 0.01).

**Table 6:** The results of stepwise multivariate regression analysis for independent risk factors associated with ACLF cases.

Indexes	AOR (95% CI) in ACLF cases
Age(years)	1.03 (1.00–1.05)^b^
Genotype B	4.62 (2.51–8.51)^a^
T216C	2.60 (1.39–4.85)^a^
G1896A	1.84 (1.03–3.28)^b^
G1913A/G	2.31 (1.32–4.02)^a^
A2159G/C	2.76 (1.56–4.87) ^a^

ACLF, acute on chronic liver failure; CI, confidence interval; AOR, adjusted odds ratio.

^a^ *P*<0.01, as compared with non-ACLF.

^b^ *P*<0.05, as compared with non-ACLF.

### The combined HBV mutations associated with ACLF and the sensitivity and specificity of HBV mutations for ACLF

The SHEsis software platform was used to estimate the combined mutations of hepatitis virus genome and mutations independently associated with ACLF were included in the analysis. Haplotype containing wild types at all of four mutation sites was inversely associated with ACLF (7.7% in ACLF VS 48.2% in non-ACLF, *P* < 0.01). Rather than a single mutation, combinations with any 2 or more of T216C, G1896A, C1913A/G and A2159G/C were significantly associated with the development of ACLF compared with non-ACLF (78.8% vs. 29.2%, *P*<0.01) (Table 7). The frequencies of C216 in any combination, A1896 in any combination, A/G1913 in any combination, and G/C2159 in any combination were 47.1%, 65.4%, 53.8%, and 42.3% in the patients with ACLF, whereas the frequencies were only 9.6%, 26.6%, 19.2% and 15.0% in those with non-ACLF (*P<*0.01). We then evaluated the potential value of the presence of HBV mutations that were independently associated with ACLF for the indication of ACLF. As shown in Table 7, Combinations with any 2 or more mutations had a moderate sensitivity and specificity for ACLF. C216 in any combination, A/G1913 in any combination, and G/C2159 in any combination had a high specificity for ACLF (90.4%, 80.8%, and 85.0%). C216 or A1896, A1896 or A/G1913 and A1896 or G/C2159 were sensitive for ACLF (83.7%, 83.7%, and 84.6%).

**Table 7:** Sensitivity and specificity of specific mutation patterns of hepatitis B virus for ACLF.

HBV mutation pattern	ACLF	Non-ACLF	Sensitivity	Specificity
	(n = 104)(%)	(n = 334)(%)	(95% CI) (%)	(95% CI) (%)
No mutation	7.7^a^	48.2	—	—
G/C2159 alone	2.9	4.5	—	—
A/G1913 alone	1.9	3.3	—	—
A1896 alone	6.7	11.1	—	—
C216 alone	1.9	3.3	—	—
Combined mutations ^b^	82 (78.8) ^a^	99(29.2)	78.8(71.0–86.0)	70.4(65.1–75.1)
C216 in any combination	49(47.1) ^a^	32(9.6)	47.1(37.5–56.7)	90.4(87.3–93.6)
A1896 in any combination	68(65.4) ^a^	89(26.6)	65.4(56.2–74.5)	73.4(68.6–78.1)
A/G1913 in any combination	56(53.8) ^a^	64(19.2)	53.8(44.3–63.4)	80.8(76.6–85.1)
G/C2159 in any combination	44(42.3) ^a^	50(15.0)	42.3(32.8–51.8)	85.0(81.2–88.9)
C216 or A896	87(83.7) ^a^	140(51.9)	83.7(76.5–90.8)	58.1(52.8–63.4)
C216 or A/G1913	80(76.9) ^a^	104(31.1)	76.9(68.8–85.0)	68.9(63.9–73.8)
C216 or G/C2159	73(70.2) ^a^	101(30.2)	70.2(61.4–79.0)	69.8(64.8–74.7)
A1896 or A/G1913	87(83.7) ^a^	146(43.7)	83.7(76.5–90.8)	56.3(51.0–61.6)
A1896 or G/C2159	88(84.6) ^a^	149(44.6)	84.6(75.9–90.7)	55.4(50.0–60.7)
A/G1913 or G/C2159	77(74.0) ^a^	112(33.5)	74.0(65.6–82.5)	66.5(61.4–71.5)

ACLF, acute on chronic liver failure; CI, confidence interval; —,not calculated.

^a^ *P*<0.01, as compared with non-ACLF.

^b^ combinations with any 2 or more of T216C, G1896A, C1913A/G and A2159G/C.

### Risk factors independently associated with the mortality of patients with ACLF

Fifty of the 104 ACLF patients survived for at least 3 months after the onset of liver failure, while 54 patients had a fatal outcome. Patients’ age, sex, HBV genotypes, HBV DNA level (log10 copies/ml), HBeAg status, ALT, TBIL, PTA, and the presence of mutations at the seven sites were included in the multivariate regression model. It was found that the higher TBIL (OR 1.006[1.002–1.009], P<0.01) and lower PTA (OR 0.002 [0.00–0.064], P<0.01) were independently risk factors of the mortality of patients with ACLF.

## Discussion

Up to now, many antiviral drugs, such as lamivudine, adefovir, Tenofovirand entecavir, have been used to treat hepatitis B, and one of the key issues for preventing the development of ACLF is early antiviral treatment of chronic hepatitis B (CHB) with acute exacerbation [17, 18]. So it is important for us to find some viral biomarkers related to hepatitis B progression and to diagnose ACLF as early as possible.

**Fig 1:**
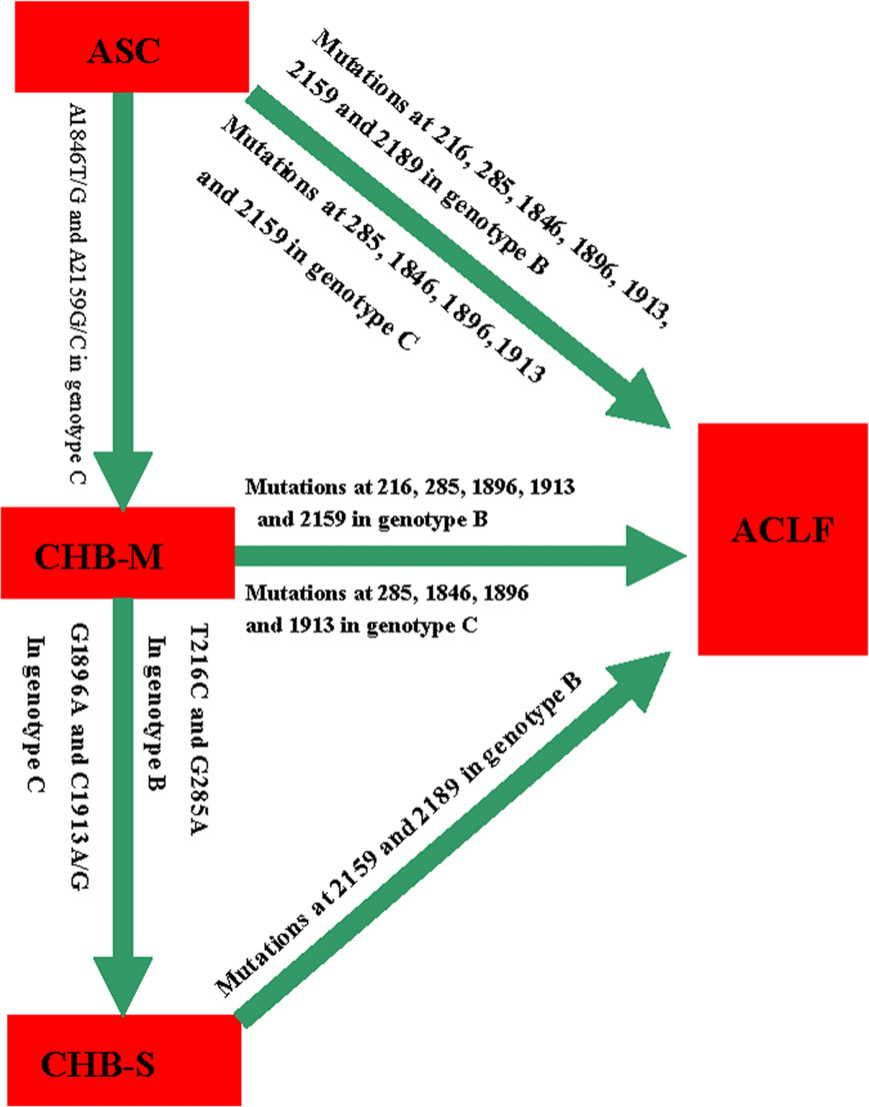
The associations of seven mutations in hepatitis B virus (HBV) with asymptomatic hepatitis B surface antigen carriers, mild chronic hepatitis B, severe chronic hepatitis B, acute on chronic liver failure in genotypes B and C.

The results of Illumina high-throughput sequencing in present study indicated that there were significant differences of the mutations at seven sites of HBV between twelve ACLF cases and twelve CHB-M cases. Of these seven mutations, A1846T/G, G1896A and C1913A/G were studied extensively, whereas T216C, G285A, A2159G, and A2189C were novel ACLF-related mutations identified in this study [6, 7]. To verify the association between the newly identified mutations of HBV and ACLF, we carried out a case control study on 80 cases with ASC, 152 cases with CHB, 102 cases with CHB-s and 104 cases with ACLF. As HBV mutations may consecutively increase with aging and the severe hepatitis are more frequent in men than in women [19, 20], we adjusted the age and sex in evaluating the association of seven mutations with ASC, CHB-M, CHB-S, or ACLF. It was found that in the subjects with genotype B, the mutations at nt.216, nt.285, nt.1896, and nt.1913 sites were significantly related to CHB-S or ACLF as compared to ASC; the mutations at nt.2159 and nt.2189 sites were significantly associated with ACLF as compared to CHB-S (Fig 1). Although the symptoms of early ACLF are similar to the symptoms of CHB-S, the prognosis of ACLF was much poorer than that of CHB-S, so A2159G/C and A2189T/C may be very important, which could serve as markers to distinguish ACLF from CHB-S in the subjects with genotype B. In the subjects with genotype C, the mutations at nt.285, nt.1846, nt.1896, and nt.1913 sites were significantly associated with ACLF as compared to the CHB-M or ASC (Fig 1). The results of present instigation prompted that these mutations, alone or in combination, may be utilized to recognize the different stages of hepatitis B.

As compared with the non-ACLF group, the AOR of T216C and A2189T/C mutation in ACLF cases with genotype B significantly increased, but AOR of T216C and A2189T/C mutation in ACLF cases with genotype C did not elevate significantly. It was suggested that HBV genotypes with distinct patterns of nucleotide substitutions were related to ACLF development. Multivariate regression analysis showed that T216C, G1896A, C1913A/G and A2159G/C mutations were independent risk factors for ACLF cases. Some previous studies also indicated that G1896A and C1913A/G mutations were in close relationship with ACLF [7]. However, the results of stepwise multivariate regression analysis in our study exhibited that AOR values of T216C and A2159G/C mutations in ACLF cases were 2.60 (1.39–4.85) and 2.76 (1.56–4.87) that significantly enhanced as compared with non-ACLF cases. So far the mutations at T216C and A2159G/C sites as independent risk factors for ACLF have not been reported. Thus, according to the results of our investigation, the old HBV patients with genotype B virus and mutations at T216C, G1896A, C1913A/G and A2159G/C sites may be prone to ACLF development.

Also it was found that the frequencies of T216C, G1896A, G1913A/G, and A2159G/C were >45% in the patients with ACLF, the combinative mutations at these sites could be utilized to diagnose clinically and to discriminate the HBV-infected patients with advanced disease. When the association of combined mutations of HBV with CHB progression was analyzed, we found that C216 in any combination, A/G1913 in any combination, and G/C2159 in any combination were specific for ACLF (Table 7). Thus combined mutations in hepatitis B cases may play more important role in indentifying ACLF than the single mutations.

Till now, the biological mechanism of CHB deterioration related to virus mutations has not been clear. Of the HBV mutations at nt.216, nt.1896, nt.1913 and nt.2159 sites independently associated with ACLF, only G1896A has been studied extensively and it can abolish HBeAg expression at the translational level [21–24]. The C1913A/G mutation causes a substitution of the fifth amino acid (proline changes to threonine or alanine) in core protein, which may alter function of HBcAg [7, 25]. The 2159 A to G/C is a missense mutation resulting in the amino acid change of HBcAg codon 87. Codon 87 is located within a known B-cell epitope and this mutation may change the immunodominant epitopes of HBcAg and promote immune clearance and liver damage [26–28]. Also T216C is a missense mutation, which can cause the amino acid changes of HBsAg codon 21. Codon 21 is located at the amino-terminal transmembrane domain between residues 4 and 24, i.e. the signal sequences of HBV envelope proteins [29]. This mutation may alter the release of virus and subviral particles, causing the direct cytotoxicity. But, these hypotheses should be determined with site-directed mutations and direct transfection experiments.

In conclusion, the associations of HBV mutations with ASC, CHB-M, CHB-S, and ACLF were investigated systemically in present study. It was found that patients with CHB infected with genotype B virus were more prone to development of ACLF than those with genotype C virus. T216C, G1896A, C1913A/G and A2159G/C were factors independently associated with ACLF. A combined examination of different viral mutations could predict more precisely the progression of liver disease. These virus mutations, as molecular biomarkers, may be useful for preventing ACLF development and predicting prognosis of patients with hepatitis B.

## Supporting Information

S1 Fig Plot of base substitution rate for the plasmid control as a function of read position. As shown in the plot, the first 20 base pairs of the read contain the lower amount of error.
(TIF)
