# Supplementary figures and images for: New Point Mutations in Surface and Core Genes of Hepatitis B Virus Associated with Acute on Chronic Liver Failure Identified by Complete Genomic Sequencing
Source: PLoS One. 2015 Apr 7;10(4):e0123139. doi: 10.1371/journal.pone.0123139 (PMC4388673; doi:10.1371/journal.pone.0123139)

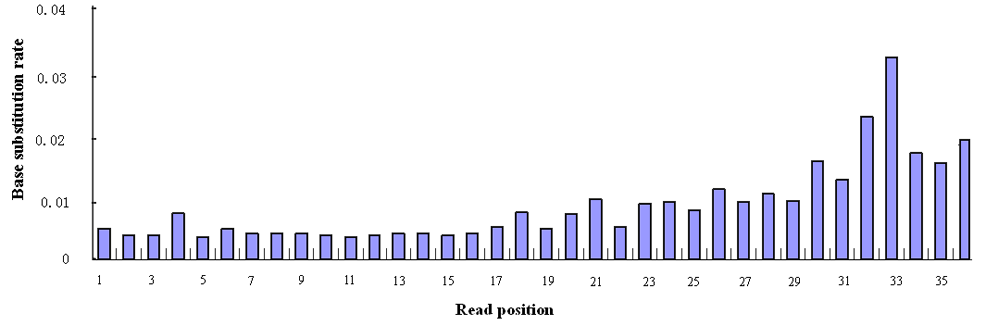

Supplement: S1 Fig — As shown in the plot, the first 20 base pairs of the read contain the lower amount of error. (TIF) [file pone.0123139.s001.tif]
